# Identification of some bioactive compounds from *Trignonella foenumgraecum* as possible inhibitors of PPAR_ϒ_ for diabetes treatment through molecular docking studies, pharmacophore modelling and ADMET profiling: An in-silico study

**DOI:** 10.1371/journal.pone.0284210

**Published:** 2023-05-18

**Authors:** Olayinka Sunday Okoh, AbdulbasitHaliru Yakubu, Abayomi Emmanuel Adegboyega, Daniel Ejim Uti, Uket Nta Obeten, Samuel Ali Agada, Folusho Oluwaloni, Grace Inioluwa Johnson, Leonard Paul Mela, Rita Onyekachukwu Asomadu, Opeyemi Iwaloye, Titilayo Omolara Johnson, Obasi Uche. Orji

**Affiliations:** 1 Department of Chemical Sciences, Anchor University, Lagos, Nigeria; 2 Department of Pharmaceutical Chemistry, University of Maiduguri, Maiduguri, Nigeria; 3 Department of Biochemistry, Faculty of Basic Medical Science, University of Jos, Jos, Nigeria; 4 Jaris Computational Biology Centre, Jos, Nigeria; 5 Department of Biochemistry, Faculty of Basic Medical Sciences, College of Medicine, Federal University of Health Sciences, Otukpo, Benue State, Nigeria; 6 Department of Chemistry/Biochemistry and Molecular Biology, Alex Ekwueme Federal University, Ndufu-Alike Ikwo, Abakaliki, Ebonyi State, Nigeria; 7 Department of Biotechnology, Federal Institute of Industrial Research, Oshodi, Lagos, Nigeria; 8 Faculty of Medical Sciences, College of Health Sciences, University of Jos, Jos, Nigeria; 9 Department of Clinical Pharmacology and Therapeutics, College of Medical Sciences, University of Maiduguri, Maiduguri, Nigeria; 10 University of Nigeria, Nnsuka, Nigeria; 11 Bioinformatics and molecular biology Unit, Department of Biochemistry, Federal University of Technology Akure, Akure, Ondo, Nigeria; 12 Department of Biochemistry, Faculty of Science, Ebonyi State University, Abakaliki, Ebonyi State, Nigeria; Gauhati University, INDIA

## Abstract

Oral antidiabetic agents including the peroxisome proliferator-activated receptor gamma (PPARγ) agonists are available for the clinical management of diabetes mellitus (DM) but most are characterized by many adverse effects. In this study, we explore the antidiabetic properties of phytoconstituents from *Trigonellafeonumgraecum (Fabaceae)* as potential agonist of PPARγ; using in silico molecular docking, molecular mechanics generalized surface area (MM/GBSA)free binding energy prediction, Pharmacophore modeling experiment, and Pharmacokinetic/ toxicity analysis. One hundred and forty (140) compounds derived from *Trigonellafeonumgraecum* were screened by molecular docking against protein target PDB 3VI8. Results obtained from binding affinity (BA) and that of binding free energy (BFE) revealed five 5 compounds; arachidonic acid (CID_10467, BA -10.029, BFE -58.9), isoquercetin (CID_5280804, BA -9.507kcal/mol, BFE -56.33), rutin (CID_5280805, BA -9.463kcal/mol, BFE -56.33), quercetin (CID_10121947, BA -11.945kcal/mol, BFE -45.89) and (2*S*)-2-[[4-methoxy-3-[(pyrene-1-carbonylamino)methyl]phenyl]methyl]butanoic acid (CID_25112371, BA -10.679kcal/mol, BFE -45.73); and were superior to the standard; Rosiglitazone with a docking score of -7.672. Hydrogen bonding was notable in the protein-ligand complex interaction, with hydrophobic bond, polar bond and pipi stacking also observed. Their Pharmacokinetic/ toxicity profile showed varying druggable characteristics, but; arachidonic acid had the most favorable characteristics. These compounds are potential agonists of PPARγ and are considered as antidiabetic agents after successful experimental validation.

## 1. Introduction

Diabetes mellitus (DM) is a chronic metabolic disorder characterized by glucose tolerance, hyperglycemia and insulin resistance, with features such as high fasting and post-prandial blood glucose concentration resulting from defective insulin secretion, insulin action, or both [1]. Around 463 million adults are currently living with diabetes, and this may increase to 578 million and 700 million by 2030 and 2045 respectively [2, 3]. It is ranked among the top ten (10) leading causes of death worldwide [4]. There are three types of diabetes: Type I Diabetes (Insulin Dependent Diabetes Mellitus), Type II Diabetes (Non-Insulin Dependent Diabetes Mellitus) and Gestational diabetes. The most common form of diabetes is Type II diabetes, constituting about 90–95% of the diabetic population [5]. It is characterized by insulin resistance, thereby resulting in hyperglycemia. Hyperglycemia, in turn, leads to glucotoxicity and long-term vascular (including peripheral and coronary artery, and cerebrovascular ailments; retinopathy, neuropathy, and nephropathy) and non-vascular complications (including infections, gastroparesis, and skin changes) of diabetes mellitus [1].

The universally accepted strategy in the management of diabetes is the administration of insulin jabs and antidiabetic prescriptions containing oral hypoglycemic drugs such as insulin secretagogues, incretin agonists, insulin sensitizers, dipeptidyl peptidase-4 inhibitors, α-glucosidase inhibitors to enhance a controlled blood glucose level [6, 7]. However, the accessibility and affordability of these; coupled with their adverse effects, such as hypoglycemia, gastrointestinal upset, and lactic acidosis, have been reported in patients on antidiabetic drug therapy, and thus, pose a risk in the management of diabetes [8]. These contribute to the immense usage of herbs and plants that have distinct array of phytochemicals; paving more interest in the administration of medicinal plants with their varying pharmacological and biological activities to manage and treat diabetes [9]. One of such antidiabetic plants widely sought after is Fenugreek (*Trigonellafoenum-graecum Linn*).

Trigonellafoenum-graecum Linn., also known as Methi in various Indian languages and Fenugreek in English, is an annual and dicotyledonous plant belonging to the Fabaceae family [10]. Compounds contained in Fenugreek include but are not limited to: trigonelline, 4-hydroxy isoleucine, galactomannans, quercetin, kaempferol, luteolin, vitexin, protodioscin, diosgenin, sotolone, calycosin, tricin, apigenin, yamogenine, gentianine, scopoletin, coumarin, and tigogenin [11–13]. The antioxidant, anti-inflammatory, hepatoprotective, anti-microbial, anticancer, immunomodulatory, gastroprotective, hypocholesterolemic, and neuroprotective effects of Fenugreek have been reported [14, 15]. The antidiabetic effect of Fenugreek has also been demonstrated [5, 16–21]. With the aid of in silicovirtual screening methods, the antidiabetic mechanism of compounds isolated from medicinal plants can be ascertained against multiple diabetes targets rapidly and cost-effectively. Examples of such targets include α-amylase, α-glucosidase, glycogen synthase kinase-3β (GSK-3β), dipeptidyl peptidase-4 (DPP-4), protein tyrosine phosphatase 1B (PTP1B), glucokinase, and peroxisome proliferator-activated receptors (PPARs).

PPARs are a group of nuclear receptors which regulate gene transcription by binding to specific DNA response elements together with the retinoid-X receptor (RXR) as heterodimers [19]. Upon ligand activation, they control the expression of genes related to lipid and glucose homeostasis [17, 22, 23], thereby serving as excellent targets for the treatment of diabetes. They exist in three subtypes: α, δ, and γ. Peroxisome proliferator-activated receptor gamma (PPARγ) consists of two isoforms; PPARγ-1 and PPARγ-2. PPARγ-1 is majorly expressed in the gut, while PPARγ-2 is abundant in the adipose tissue where it is involved in adipocyte proliferation and differentiation. PPARγ agonist like the thiazolidinediones (TZD) exert their action by fully stabilizing the AF2 (activation function 2) of the ligand-binding domain (LBD), in its active conformation, thereby activating the PPARγ receptor, which in turn improves insulin sensitivity [22, 24, 25].

The present study screened and evaluated phytoconstituents obtained from Trigonellafoenum-graecum as a potential agonist of PPARγ for the treatment of diabetes mellitus via an *insilico*computational approach.

## 2. Materials and methods

### 2.1 Protein preparation

The crystal structure of peroxisome proliferator-activated receptor gamma (PPARγ) (PDB ID: 3VI8) was retrieved from Protein Data Bank (PDB) repository. The protein was prepared using the protein preparation wizard panel of Glide (Schrödinger Suite 2020–3) [26–28] where bond orders were assigned, hydrogen added, disulfide bonds created, while missing side chains and loops were filled using prime. Water molecules beyond 3.0 Å of the heteroatoms were removed and the structure was minimized using OPLS2005 and optimized using PROPKA [29, 30]. Subsequently, the receptor grid file was generated to define the binding pocket for the ligands.

### 2.2 Ligand preparation

One hundred and forty (140) compounds from *Trigonellafeonumgraecum*, obtained by gas chromatography mass spectrometry (GCMS) analysis as earlier described [31] were prepared for molecular docking using Ligprep module (Schrödinger Suite 2020–3) [32]. Low-energy 3D structures with correct chiralities were generated. The possible ionization states for each ligand structure were generated at a physiological pH of 7.2 ± 0.2. Stereoisomers of each ligand were computed by retaining specified chiralities while others were varied.

### 2.3 Receptor grid generation

Receptor grid generation allows defining the position and size of the protein’s active site for ligand docking. The scoring grid was defined based on the co-crystalized ligand (2S)-2-(4-methoxy-3-{[(pyren-1-ylcarbonyl)amino]methyl}benzyl)butanoic acid with Unique ID 13M; Chenusing the receptor grid generation tool of Schrödinger Maestro 12.5. the van der Waals (vdW) radius scaling factor of nonpolar receptor atoms were scaled at 1.0, with a partial charge cut off of 0.25.

### 2.4 Protein-ligand docking

Glide [26–28] tool of Schrödinger Maestro 12.5 [33] was used to perform the molecular docking studies using the generated receptor grid file. The prepared ligands were docked using standard precision (SP), setting ligand sampling to flexible, with the ligand sampling set to none (refine only). The vdW radius scaling factor was scaled at 0.80 with a partial charge cut-off of 0.15 for ligand atoms.

### 2.5 Receptor-ligand complex pharmacophore modelling

The first three compounds ranked with highest binding affinity against the target protein was used to develop a receptor-ligand complex pharmacophore model using PHASE [34, 35]. Auto (E-pharmacophore) method was used, hypothesis was set with maximum number of features to be generated at 7, minimum feature-feature distance at 2.00, minimum feature-feature distance for feature of the same type at 4.00 and donors as vectors.

### 2.6 Pharmacology parameters

The absorption, distribution, metabolism, excretion and toxicity (ADMET) properties of the test compounds were determined using in silico integrative model predictions at the SwissADME and PROTOX-II server respectively.

## 3. Results

### 3.1 Virtual screening of compounds

About one hundred and forty (140) compounds derived from *Trignonella foenum graecum* (Table 1) were screened against 3VI8, in which five (5) top posed compounds exhibited the highest binding affinities against the protein target and were selected for post docking analysis. From the multiple screening analysis, the following compounds arachidonic acid (CID_10467), isoquercetin (CID_5280804), rutin (CID_5280805), quercetin (CID_10121947) and (2*S*)-2-[[4-methoxy-3-[(pyrene-1-carbonylamino)methyl]phenyl]methyl]butanoic acid (CID_25112371) showed the lowest docking scores (highest binding affinity scores) of -10.029, -9.507, -9.463, -11.945 and -10.679 respectively against PDB 3VI8. This virtual screening based on binding energy gave us a vivid idea of the best ligands having the highest affinity for PDB 3VI8.

**Table 1:** Dockingscores of screened compounds derived from *Trignonella foenum graecum* against Peroxisome proliferator-activated receptor-γ.

PubChemID	Docking score	PubChemID	Docking score	PubChemID	Docking score	PubChemID	Docking score	PubChemID	Docking score	PubChemID	Docking score
**10121947**	-11.945*	**114776**	-7.811	**7009**	-6.643	20210	-5.999	27457	-5.295	305	-3.939
**25112371**	-10.679*	**11349**	-7.8	**318040061**	-6.643	82000	-5.982	5960	-5.275	1054	-3.918
**10467**	-10.029*	**5991**	-7.772	**13644663**	-6.617	971	-5.982	444170	-5.273	602	-3.887
**5280804**	-9.507*	**6305**	-7.712	**6506665**	-6.601	6918391	-5.924	44259083	-5.154	6322	-3.722
**5280805**	-9.463*	**10440782**	-7.685	**5280445**	-6.592	61700	-5.91	1054	-5.126	985	-3.711
**493570**	-9.297	**5280934**	-7.653	**442664**	-6.55	135191	-5.875	595	-5.004	595	-3.498
**5280805**	-9.043	**11727586**	-7.618	**6140**	-6.538	5281675	-5.851	119	-4.935	133082395	-3.387
**439242**	-8.975	**12267346**	-7.535	**5280863**	-6.504	366	-5.845	26331	-4.927	6274	-3.385
**5280441**	-8.58	**54675850**	-7.521	**92871**	-6.5	133082395	-5.805	156784	-4.91	12304449	-3.349
**5280343**	-8.553	**135398658**	-7.493	**323**	-6.493	6274	-5.804	5951	-4.823	750	-3.207
**5280863**	-8.519	**114776**	-7.438	**936**	-6.48	335	-5.794	6288	-4.801	938	-3.102
**318040061**	-8.477	**6029**	-7.411	**637542**	-6.399	17898	-5.794	7976	-4.762	442630	-2.581
**13644663**	-8.332	**101536190**	-7.288	**354616**	-6.327	162350	-5.695	33032	-4.722	131752196	-1.631
**5280961**	-8.229	**12306047**	-7.257	**5280961**	-6.26	11451065	-5.691	7009	-4.68	11006	-0.638
**162350**	-8.215	**938**	-7.201	**6287**	-6.246	25915	-5.685	6306	-4.659	131752196	-0.037
**5281675**	-8.052	**1130**	-7.11	**23318480**	-6.166	7938	-5.68	2773624	-4.631		
**5280378**	-8.021	**11349**	-7.091	**10329**	-6.145	131752196	-5.489	9261	-4.486		
**5280445**	-8.019	**9828626**	-7.055	**6057**	-6.14	18950	-5.442	6106	-4.441		
**5280804**	-7.992	**135398658**	-6.95	**5570**	-6.135	7361	-5.433	337494	-4.429		
**729859**	-7.937	**6325460**	-6.945	**54670067**	-6.133	6274	-5.419	971	-4.388		
**445639**	-7.906	**5281**	-6.933	**145742**	-6.105	31252	-5.403	91439	-4.333		
**439336**	-7.827	**892**	-6.854	**5280441**	-6.093	520098	-5.395	99512	-4.295		
**87508015**	-7.818	**44259083**	-6.767	**439195**	-6.082	5280378	-5.354	1130	-4.256		
**131752658**	-7.814	**442664**	-6.763	**135398658**	-6.067	1054	-5.336	62835	-4.232		
**171548**	-7.813	**27209**	-6.716	**62835**	-6.014	133082395	-5.317	5962	-4.17		

*Indicating topposed compounds with lowest binding energies (Highest binding affinities)

### 3.2 Molecular mechanics generalized surface area (MM/GBSA)

The Molecular mechanics generalized surface area (MM/GBSA) has been more accurate in binding free energy calculation studies and was used in determining the binding free energies of these compounds after virtual screening (multi-ligands docking). These best five (5) posed compounds exhibited lowest binding free energy of; -58.9 (CID_10467), -56.33 (CID_5280804), -56.33 (CID_5280805), -45.89 (CID_10121947) and-45.73 (CID_25112371) respectively with PDB 3VI8 (Table 2).

**Table 2:** Molecular mechanics generalized surface area (MM/GBSA) binding free energies of screened compounds derived from *Trignonella foenum graecum* against Peroxisome proliferator-activatedreceptor-γ.

PubChemID	BFE	PubChemID	BFE	PubChemID	BFE	PubChemID	BFE	PubChemID	BFE	PubChemID	BFE
**10121947**	-58.9*	11349	-34.46	7009	-36.34	20210	-24.25	27457	-22.79	305	-14.64
**25112371**	-56.33*	5991	-35.48	318040061	-40.36	82000	-28.32	5960	-20.75	1054	-20.84
**10467**	-41.8*	6305	-36.45	13644663	-43.79	971	-14.72	444170	-28.83	602	-17.58
**5280804**	-45.89*	10440782	-34.84	6506665	-46.44	6918391	-20.36	44259083	-47.42	6322	-27.09
**5280805**	-45.73*	5280934	-44.97	5280445	-36.87	61700	-22.4	1054	-22.24	985	-45.67
**493570**	-40.54	11727586	-21.09	442664	-49.77	135191	-17.19	595	-35.96	595	-35.33
**5280805**	-42.53	12267346	-31.79	6140	-30.97	5281675	-33.4	119	-18.56	133082395	-26.27
**439242**	-25.73	54675850	-19.7	5280863	-35.97	366	-33.97	26331	-21.4	6274	-24.42
**5280441**	-39.59	135398658	-43.93	92871	-18.53	133082395	-25.34	156784	-36.22	12304449	-20.99
**5280343**	-36.26	114776	-41.32	323	-24.05	6274	-25.34	5951	-17.97	750	-15.22
**5280863**	-36.2	6029	-34.2	936	-19.07	335	-16.61	6288	-18.55	938	-19.22
**318040061**	-40.63	101536190	-48.29	637542	-28.02	17898	-26.97	7976	-20	442630	-34.23
**13644663**	-34.57	12306047	-26.5	354616	-26.84	162350	-45.31	33032	-24.25	131752196	-23.39
**5280961**	-39.73	938	-20.45	5280961	-39.67	11451065	-46.42	7009	-36.28	11006	-41.93
**162350**	-47.31	1130	-30.29	6287	-11.89	25915	-20.7	6306	-22.04	131752196	-41.33
**5281675**	-32.04	11349	-36.31	23318480	-25.83	7938	-17.43	2773624	-17.95	14257	-30.63
**5280378**	-39.51	9828626	-51.01	10329	-20.25	131752196	-40.69	9261	-17.52		
**5280445**	-38.62	135398658	-60.85	6057	-28.76	18950	-19.6	6106	-19.61		
**5280804**	-54.99	6325460	-27.83	5570	-22.22	7361	-17.99	337494	-18.49		
**729859**	-43.08	5281	-46.13	54670067	-26.38	6274	-27.34	971	-15.09		
**445639**	-43.45	892	-19.33	145742	-19.3	31252	-20.97	91439	-18.76		
**439336**	-44.06	44259083	-38.98	5280441	-32.19	520098	-29.66	99512	-18.77		
**87508015**	-44.78	442664	-39.4	439195	-17.95	5280378	-39.13	1130	-37.35		
**131752658**	-27.05	27209	-21.07	135398658	-32.4	1054	-23.8	62835	-22.13		
**171548**	-33.42	5280343	-38.14	62835	-17.08	133082395	-28.15	5962	-26.29		

*Indicating topposed compounds with lowest binding free energies; BFE-binding free energy

### 3.3 MM/GBSA dG binding energy versus docking scores

**Fig 1:**
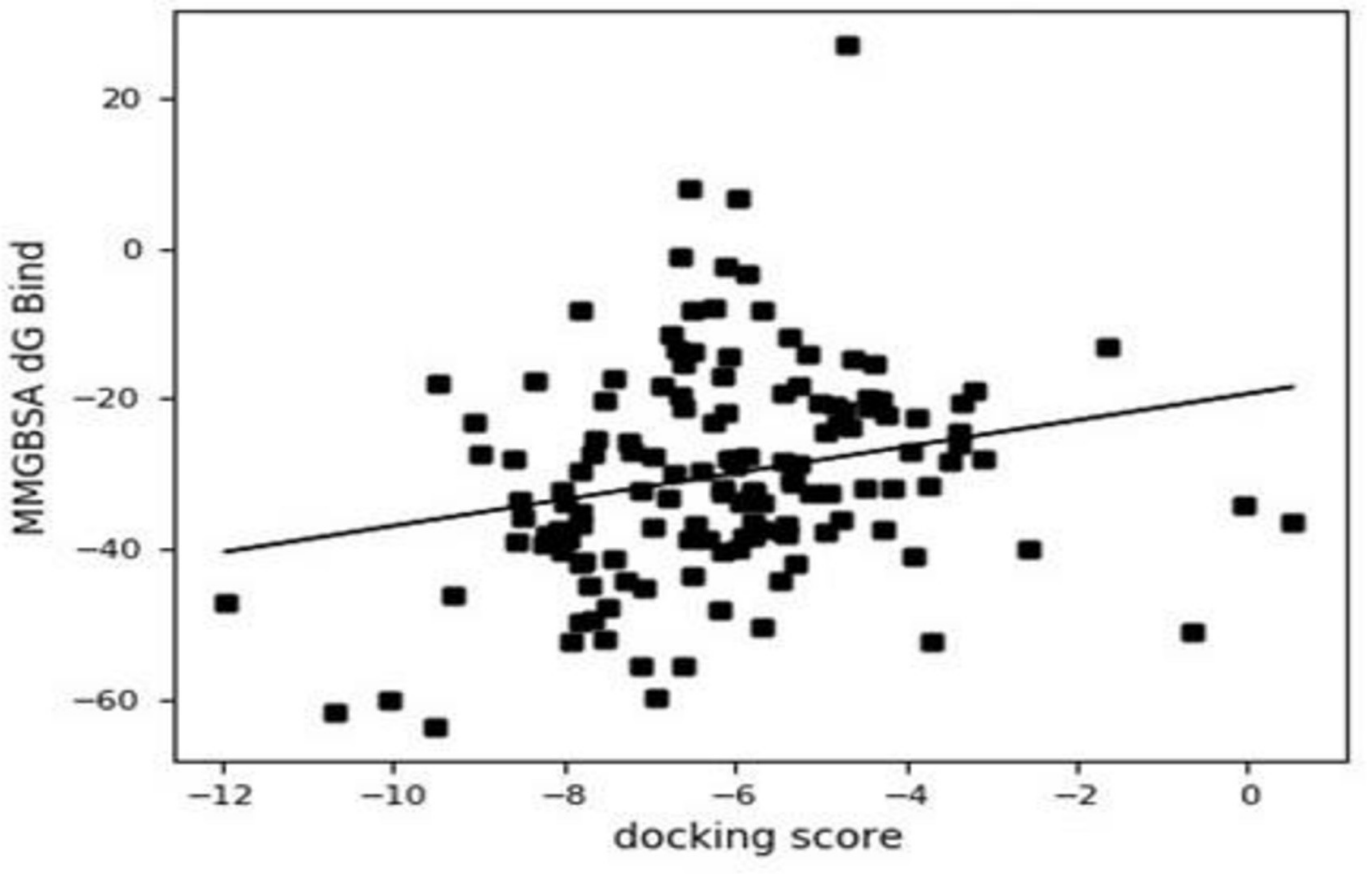
MMGBSA dG vs. docking scores.

A plot of MM/GBSA dG binding energy versus docking scores is shown in Fig 1. The graph exhibited a strong correlation between MM/GBSA dG binding energy and docking scores of the binding affinities earlier observed in the multiple docking/virtual screening analysis. Lower binding free energies of top posed compounds.

### 3.4 Molecular interactions (2D and 3D) analysis

**Fig 2:**
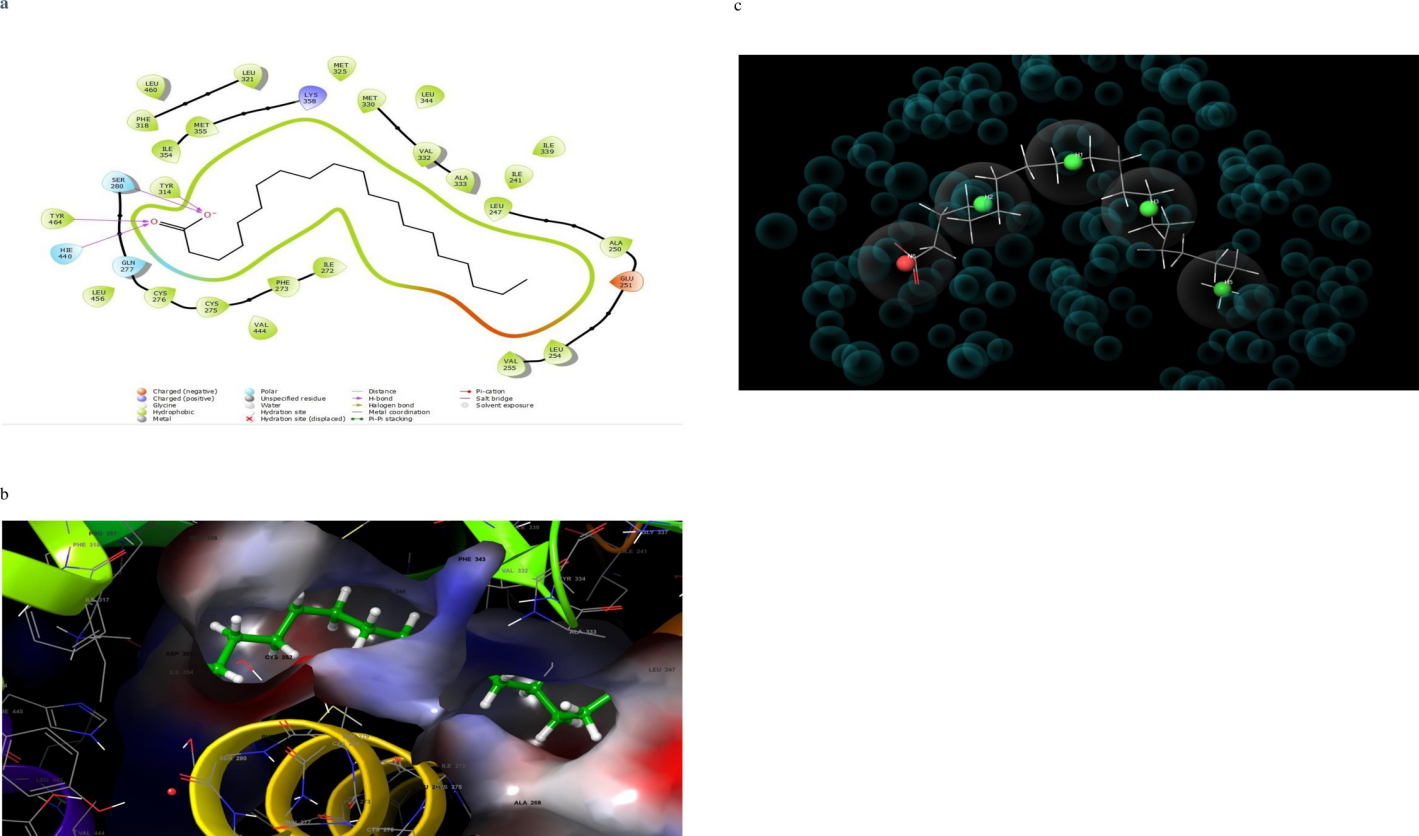
a: 2D configuration of arachidic acid complex with peroxisome proliferator-activated receptor gamma, b: 3D configuration of arachidic acid complex with peroxisome proliferator-activated receptor gamma, c: Parmacophore model of arachidic acid complex with peroxisome proliferator-activated receptor gamma.

**Fig 3:**
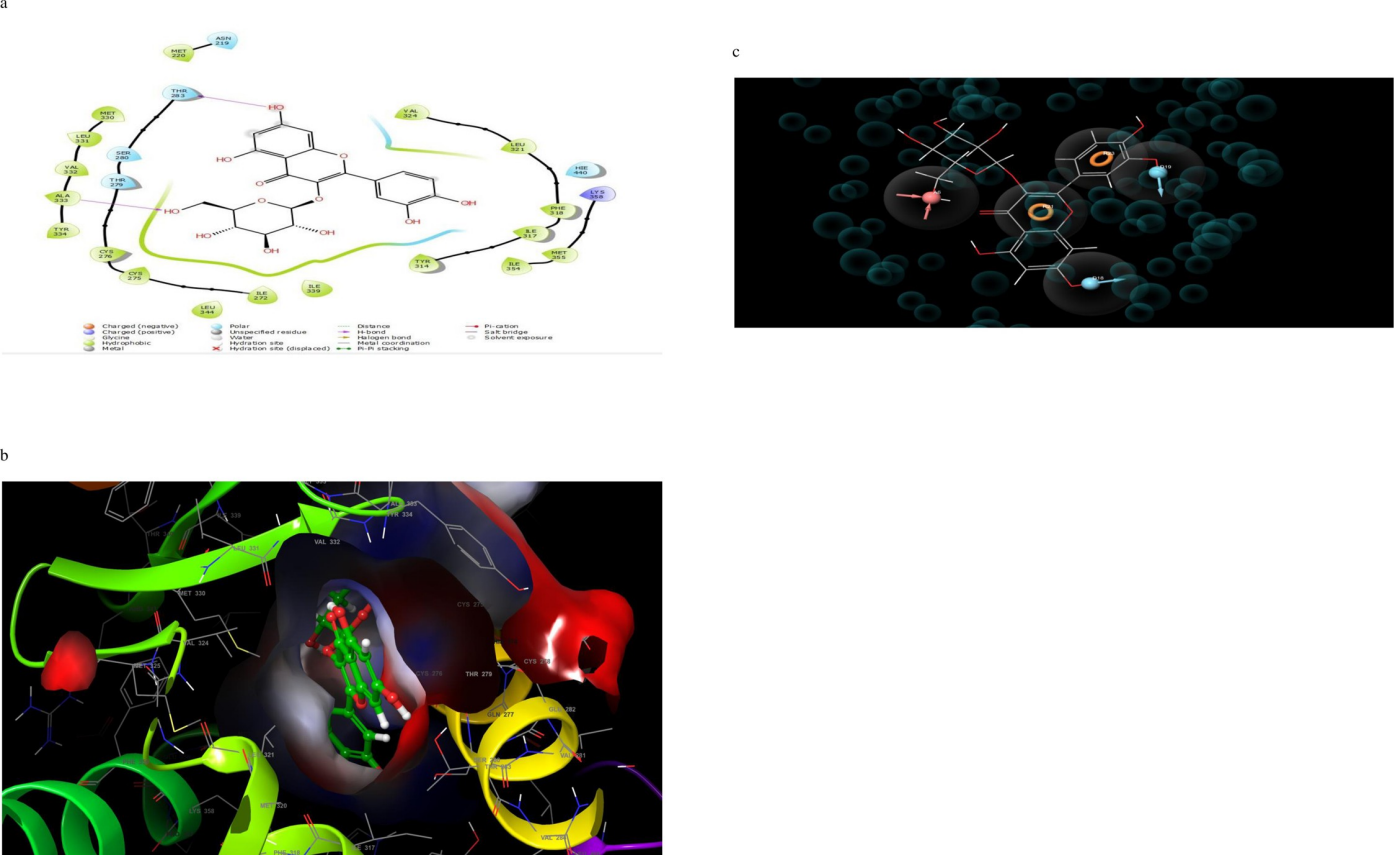
a: 2D configuration of isoquercitrin complex with peroxisome proliferator-activated receptor gamma, b: 3D configuration of isoquercitrin complex with peroxisome proliferator-activated receptor gamma, c: Parmacophore model of isoquercitrin indicating pharmacophoric sites in the ligand.

**Fig 4:**
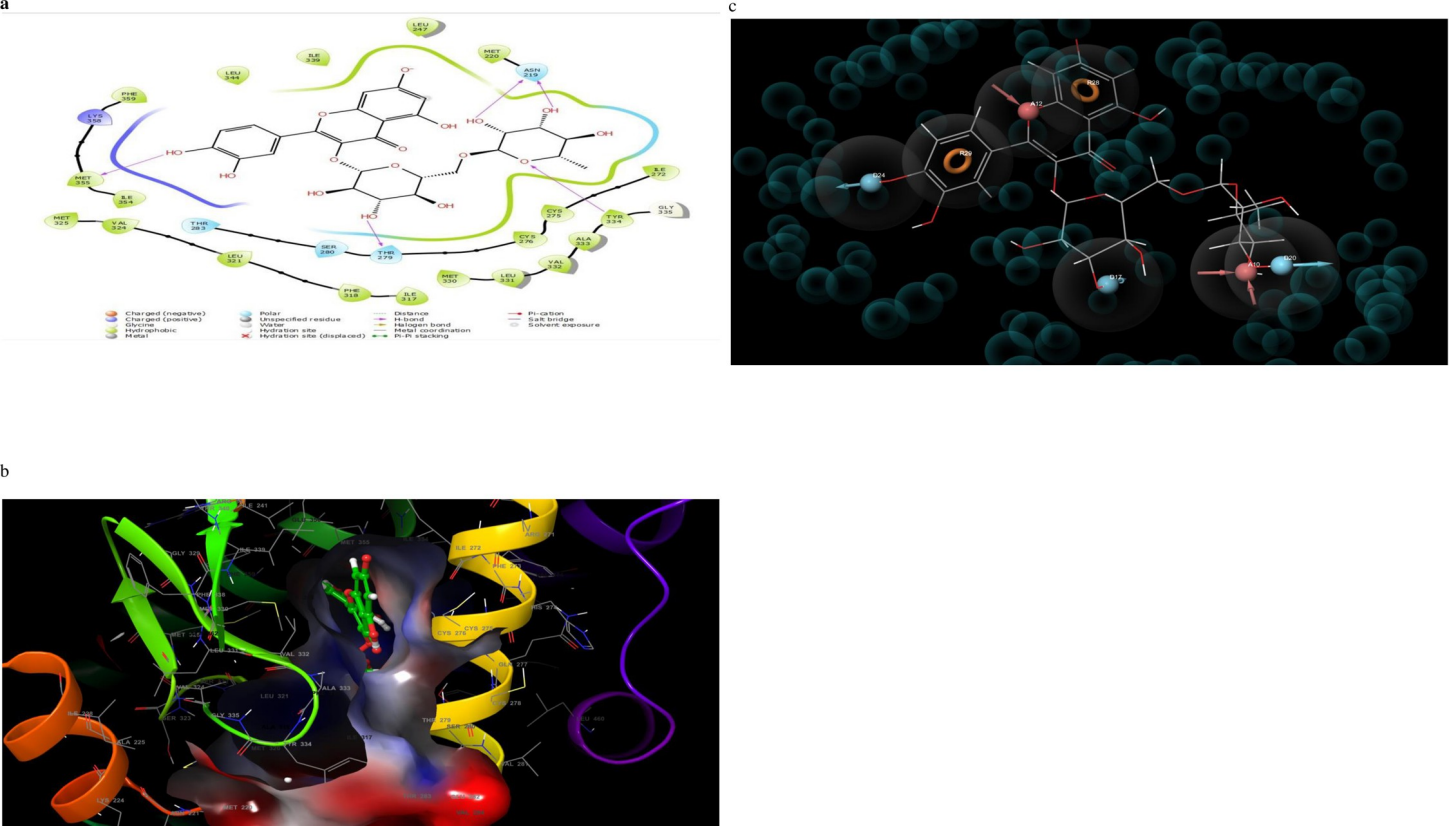
a: 2D configuration Rutin complex with peroxisome proliferator-activated receptor gamma, b: 3D configuration Rutin complex with peroxisome proliferator-activated receptor gamma, c: Parmacophore model of rutin complexes with peroxisome proliferator-activated receptor gamma.

**Fig 5:**
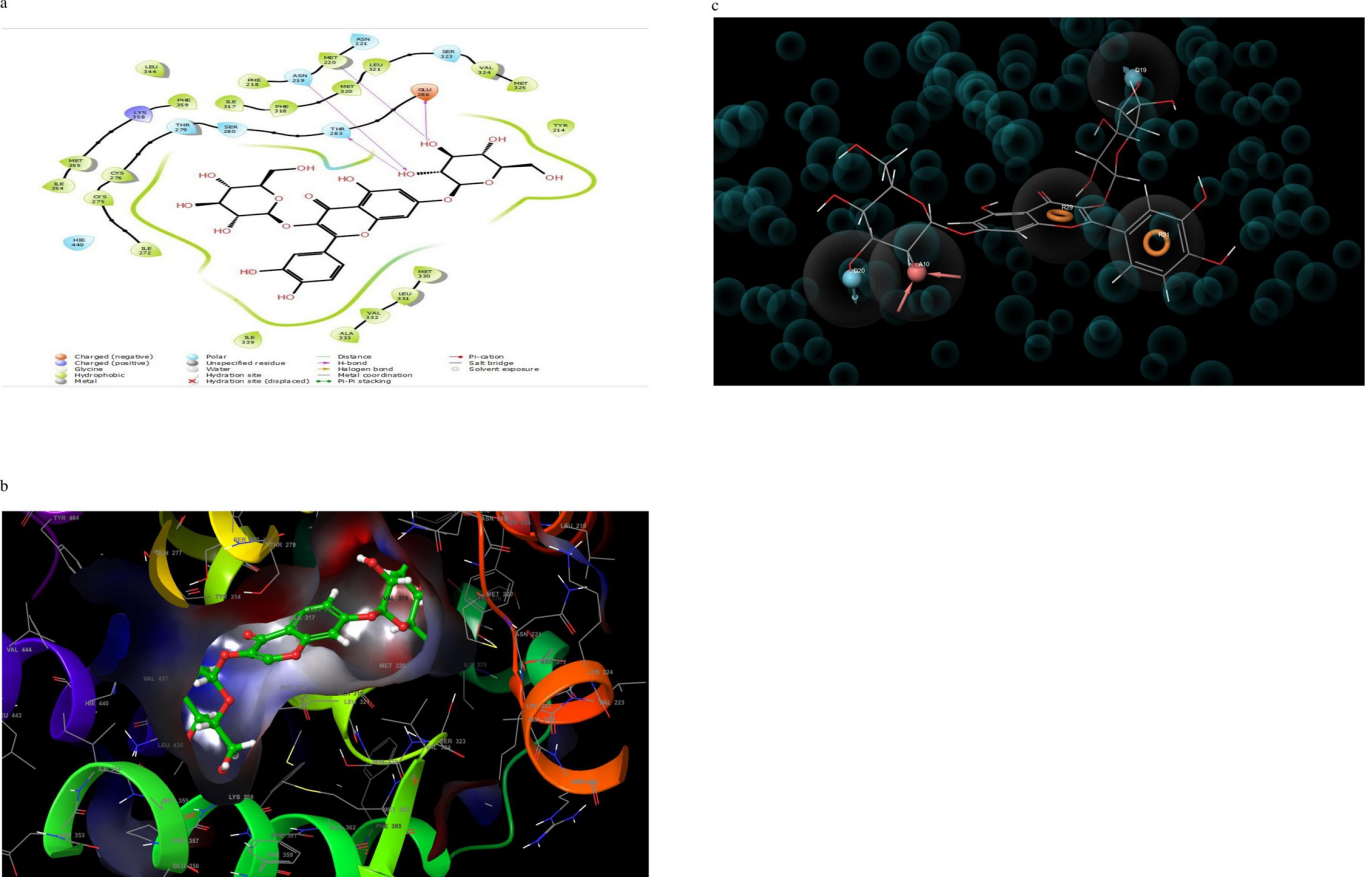
a: 2D configuration of Quercetin complex with peroxisome proliferator-activated receptor gamma, b: 3D configuration of Quercetin complex with peroxisome proliferator-activated receptor gamma, c: Parmacophore model of quercetin complex with peroxisome proliferator-activated receptor gamma.

**Fig 6:**
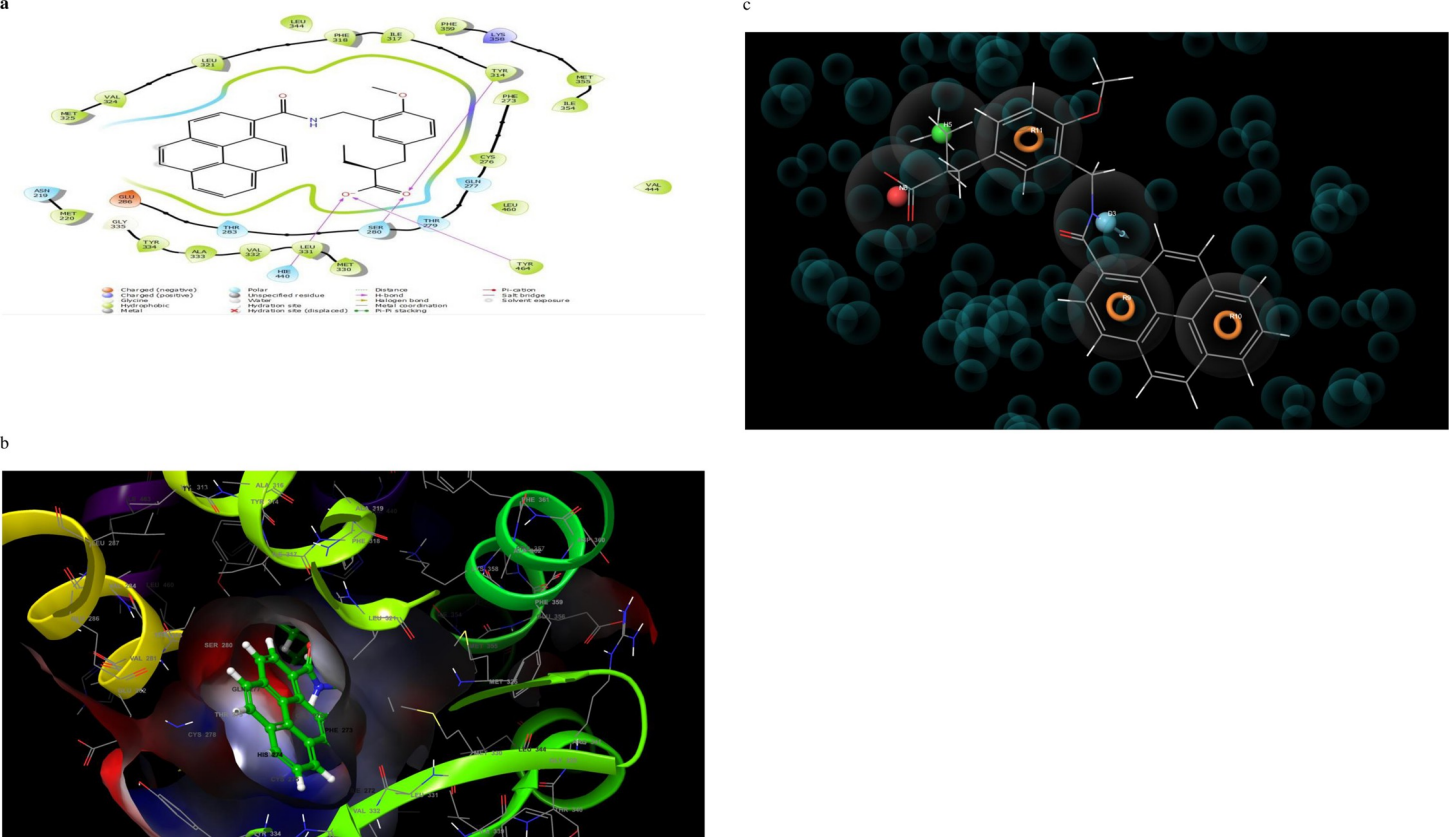
a: 2D configuration of (2s)-2-(4-Methoxy-3-{[(Pyren-1-Ylcarbonyl)amino]methyl}benzyl)butanoic Acid complex with peroxisome proliferator-activated receptor gamma, b: 3D configuration of (2s)-2-(4-Methoxy-3-{[(Pyren-1-Ylcarbonyl)amino]methyl}benzyl)butanoic Acid complex with peroxisome proliferator-activated receptor gamma, c: Parmacophore model of (2s)-2-(4-Methoxy-3-{[(Pyren-1-Ylcarbonyl)amino]methyl}benzyl)butanoic Acid complexes with peroxisome proliferator-activated receptor gamma.

Molecular interaction of the post docking studies of the ligands CID_10467, CID_5280804, CID_5280805, CID_10121947, and CID_25112371 with 3VI8 displayed well defined binding pockets constituted of residues as indicated by their 2D and 3D configuration. Threonine-314, Serine-280, Glutamine-277, Histidine-440, and Tyrosine-464 in CID_10467 ligands exhibiting hydrogen bonding to PDB 3VI8. There were also other bonds such as hydrophobic, polar, and π- π stacking present in these interactions, emphasizing the cause of the observed low binding energy (high binding affinity) between CID_10467 and PDB 3VI8 (Fig 2A and 2B). More so, the ligand CID_25112371 showed hydrogen bonding linked to the residues Histidine-440, tyrosine-464, Serine-280, and Tyrosine-314 alongside other bonds interactions with PDB 3VI8 (Fig 3A and 3B). In the ligand CID_10121947, a hydrogen bond was observed in the ligands Asparagine-219, Methionine-220, Threonine-283, and Glutamate-286 also with other bonds interactions (Fig 4A and 4B). Similarly, the ligands CID_5280804 and CID_5280805 also showed hydrogen bonds between residues Threonine-283, Alanine-333, and Tyrosine-334, Threonine-279, Asparagine-219, and Methionine-355 respectively alongside other bonds interactions with the protein target PDB 3VI8 (Figs 5A and 5B and 6A and 6B). Overall, these bonding interactions corroborated with the observed binding affinities and binding free energies between the ligands and the protein target.

### 3.5 Development of pharmacophore hypothesis for the top posed ligands

The top-five pharmacophore hypotheses were generated using these set ligands (Table 3). These hypotheses could be classified into three (3) groups according to the features in the pharmacophore model ADDRR-10121947, ADDRR-5280804, AADDDRR-5280805 (01 and 04 and 05), DHNRRR-25112371 (02), and HHHHN-10467 (03) (Figs 2C, 3C, 4C, 5C and 6C). The set hypotheses were classified by their locations and the direction of their features.

**Table 3:** Summary of the pharmacophore models for PPAR-γ agonists.

Ligand	Pubchem ID	Pharmacophore model
**Arachidonic acid**	10467	**HHHHN**
**Isoquercetin**	5280804	**ADDRR**
**Rutin**	5280805	**AADDDRR**
**Quercetin**	10121947	**ADDRR**
**(2*S*)-2-[[4-methoxy-3-[(pyrene-1-carbonylamino) methyl]phenyl]methyl]butanoic acid**	**25112371**	**DHNRRR**

H-hydrophobic group; A-hydrogen bond acceptor; R-ring-aromatic; N-negative charged group

### 3.6 Absorption Distribution Metabolism Excretion & Toxicity (ADMET)/Pharmacokinetics predictions analysis of top posed compounds

The analysis of three lead compounds using the Lipinski rule of five, Ghose, and Verber rule is presented in Table 4 for its drug-likeness, while the absorption and metabolism profiles of these compounds are presented in Tables 5 and 6 with the toxicity profile given in Table 7. There were different degrees of violation of the three (3) rules for drug-like properties as indicated especially on the H-bond donors, H-bond Acceptors, and molecular weight. It was only CID_25112371 that showed high human gastrointestinal tract absorption and bioavailability with CID_10121947 and CID_10467 exhibiting low GIT absorption profiles. These ligands also exhibited varying degrees of cytochrome–P variants inhibition profiles with also different toxicological properties towards certain target organs and features.

**Table 4:** Drug-likeness profile of leads compounds from *Trignonella foenum graecum*.

Compound	PubChem ID	Mol. wt.	iLogP	HBD	HBA	Lipinski Violation	Ghose violation	Verber violation
**Quercetin**	10121947	626.52	2.06	11	17	3	4	**1**
**(2*S*)-2-[[4-methoxy-3-[(pyrene-1- carbonylamino) methyl]phenyl]met hyl]butanoic acid**	25112371	465.54	3.8	2	4	1	2	**0**
**Arachidonic acid**	**10467**	**312.53**	**4.56**	**1**	**2**	**1**	**1**	**1**

HBD—H-bond Donors; HBA—H-bond Acceptors. Mol. Wt–molecular weight

**Table 5:** Absorption profile of leads compounds from *Trignonella foenum graecum*.

Compound	PubChem ID	Mol. wt.	BBB	HGIA	P-G S
Quercetin	10121947	626.52	No	Low	**Yes**
(2*S*)-2-[[4-methoxy-3-[(pyrene-1- carbonylamino) methyl]phenyl]met hyl]butanoic acid	25112371	465.54	No	High	**Yes**
Arachidonic acid	**10467**	**312.53**	**No**	**Low**	**No**

HGIA—Human Gastro-Intestinal Absorption BBB—Blood-Brain Barrier P-GS P-Glycoprotein Substrate

**Table 6:** Metabolism profile of leads compounds from *Trignonella foenum graecum*.

Compound	PubChem ID	Mol. wt.	CYP1A2 inhibitor	CYP2C19 inhibitor	CYP2C9 inhibitor	CYP2D6 inhibitor	CYP3A4 inhibitor
Quercetin	10121947	626.52	No	No	No	No	**No**
(2*S*)-2-[[4-methoxy-3-[(pyrene-1 carbonylamino) methyl]phenyl]methy l]butanoic acid	25112371	465.54	No	Yes	No	Yes	**Yes**
Arachidonic acid	**10467**	**312.53**	**Yes**	**No**	**No**	**No**	**No**

**Table 7:** Toxicity profile of leads compounds from *Trignonella foenum graecum*.

S/No	Target feature	Ligand-10121947	Ligand-25112371	Ligand-10467
1	Hepatotoxicity	Inactive	Inactive	Inactive
2	Carcinogenicity	Inactive	Inactive	Inactive
3	Immunotoxicity	Active	Active	Inactive
4	Mutagenicity	Inactive	Inactive	Inactive
5	Cytotoxicity	Inactive	Inactive	Inactive
6	Aryl hydrocarbon Receptor (AhR)	Inactive	Inactive	Inactive
7	Androgen Receptor (AR)	Inactive	Inactive	Inactive
8	Androgen Receptor Ligand Binding Domain (AR-LBD)	Inactive	Inactive	Inactive
9	Aromatase	Inactive	Inactive	Inactive
10	Estrogen Receptor Alpha (ER)	Inactive	Inactive	Inactive
11	Estrogen Receptor Ligand Binding Domain (ER-LBD)	Inactive	Inactive	Inactive
12	Peroxisome Proliferator Activated Receptor Gamma (PPAR-Gamma)	Inactive	Inactive	Inactive
13	Nuclear factor (erythroid-derived 2)-like 2/antioxidant responsive element (nrf2/ARE)	Inactive	Inactive	Inactive
14	Heat shock factor response element (HSE)	Inactive	Inactive	Inactive
15	Mitochondrial Membrane Potential (MMP)	Inactive	Inactive	Inactive
16	Phosphoprotein (Tumor Supressor) p53	Inactive	Inactive	Inactive
17	ATPase family AAA domain-containing protein 5 (ATAD5)	Inactive	Inactive	Inactive
18	Predicted LD50	5000mg/kg	1420mg/kg	900mg/kg
19	Predicted Toxicity Class	5	4	4

## 4. Discussion

The search for more effective and safer therapeutic alternatives is a noble task. This is necessary to reduce DM-related complications, morbidity, and mortality resulting from multisystem defects. Natural products provide an inexhaustible pool of pharmacologically active compounds with therapeutic potentials in DM treatment. Scientific screening of the vast number of natural compounds against important molecular targets for DM is an enormous task going by the conventional methods of drug development. Recent advances such as the in-silico molecular docking and ADME(T) techniques provide an invaluable tool for time- and resource-efficient screening of potential therapeutic compounds.

In the present study, a total of one hundred and forty (140) compounds derived from *Trigonellafeonumgraecum* were screened by molecular docking against peroxisome proliferator-activated receptor gamma (PPAR_ϒ_) for potential PPAR_ϒ_agonistic action. Out of the 140 screened compounds, 5 (namely; arachidonic acid, quercetin, isoquercetin, rutin, and (2*S*)-2-[[4-methoxy-3-[(pyrene-1-carbonylamino) methyl]phenyl]methyl]butanoic acid) emerged as top pose compounds, whilst 3 compounds (arachidonic acid, quercetin and (2*S*)-2-[[4-methoxy-3-[(pyrene-1-carbonylamino) methyl]phenyl]methyl]butanoic acid) showed the most drug-like characteristics and overall; arachidonic acid had the most favorable characteristics. Some molecular docking studies reported Trigonelline and Diosgenin as top pose compounds of *Trigonellafoecumgraecum* with the strongest interactions against PPAR_ϒ_ [5, 17, 19]_._ The reports showed that Trigonelline had a moderate affinity for the target while Diosgenin was better [5, 17, 19, 21]. The present study however revealed that arachidonic acid, 2S]… butanoic acid and quercetin are the ligands with superior drug-like characteristics. Trigonelline only showed a moderate binding affinity (docking score of -6.135) whereas Diosgenin had no docking score in the present study. Arachidonic acid had a docking score of -10.029 which indicates a strong affinity for the target protein (PPAR_ϒ_). This binding affinity is superior to that of Rosiglitazone (a standard PPAR_ϒ_ agonist) which showed a docking score of -7.672 in the present study. It’s also superior to Rosiglitazone’s docking score of -7.176 reported by Tharaheswari *et al*. [17]. In addition, arachidonic acid formed a strong bond with the target with binding free energy (BFE) of -58.9. The docking score and BFE value denote superior interaction and binding efficiency which suggest that arachidonic acid is potentially a superior PPAR_ϒ_ agonist to Rosiglitazone. Earlier studies suggested that arachidonic acid is an endogenous agonist of the α-subunit of PPARs whereas polyunsaturated fatty acids (PUFA) and PGI_2_ are endogenous ligands of the ϒ-subunit [36, 37]. This study revealed that arachidonic acid is also a strong agonist of the ϒ-subunit. Agents that activate PPAR_ϒ_ or enhance the expression of the PPAR_ϒ_ genes have been associated with enhancement of small insulin-sensitive adipocytes, uptake and storage of fats by adipose tissues, reduction of lipotoxicity on pancreatic β cells and other insulin-sensitive cells, and increased insulin sensitivity of liver and muscle cells [38–41]. The post docking analysis revealed hydrogen bond was (mainly) involved in ligands interaction with PPAR_ϒ_. Arachidonic acid interacted with the target amino acid residues of Thr 314, Ser 240, Glu 277, His 440, and Tyr 464. Threonine, Serine, and Tyrosine residues confer favorable flexibility to the ligand-protein complex due to their rotatable OH groups [42]. Additional bonds in the interaction included hydrophobic bond, polar bond, and pipi stacking. The detailed druggable characteristics of arachidonic acid ligand revealed in the ADMET analysis are shown in Table 4. Other notable characteristics include strong compliance to Lipinski, Ghose, and Verber’s rules, non-inhibition of important drug-metabolizing enzymes (CYP 2C19, CYP 2D6, CYP 3A4, and CYP 2C9), and low potential for organ toxicity (toxic class 4, Table 7). The post docking analyses also revealed that the other four (4) top pose compounds had characteristics similar to arachidonic acid (Tables 1–7). Their interaction with the target (PPAR_ϒ_) was also due to the hydrogen bond (mainly) with little contributions of other bonds. They showed a less efficient bond with the target compared to arachidonic acid, except for quercetin which is better only with a binding affinity (docking score -11.945). However, their docking scores were all superior to that of Rosiglitazone (-7.672). The docking scores were -11.945, -10.679, -9.507 and -9.467, and BFEs were -45.89, -45.73, -56.33 and -56.33 for quercetin, 2S]… butanoic acid, isoquercetin and rutin respectively (Tables 2 and 3). The ADMET properties of these compounds showed that they have variable oral absorptivity, could inhibit some CYP enzymes and interfere with other drug’s metabolism, and are potentially immunotoxic. Their other druggable features and ADMET properties are shown in the Tables 3–6.

## 5. Conclusion

Overall, the present study and post docking analyses revealed potential antidiabetic compounds not commonly reported in the screening of *Trigonellafoenumgraecum*. The compounds showed superior binding affinity and bond strength compared to the standard PPAR_ϒ_ agonist (rosiglitazone) and are therefore potentially more efficacious. Their pure forms need to be synthesized and tested in *invivo* studies to validate their efficacy and safety in diabetics. These could emerge as novel therapeutic agents of choice in DM treatment.

## Supporting information

S1 Graphical abstract (DOCX)

S1 Raw data (RAR)
