# Supplementary figures and images for: Identification of some bioactive compounds from Trignonella foenumgraecum as possible inhibitors of PPARϒ for diabetes treatment through molecular docking studies, pharmacophore modelling and ADMET profiling: An in-silico study
Source: PLoS One. 2023 May 18;18(5):e0284210. doi: 10.1371/journal.pone.0284210 (PMC10194899; doi:10.1371/journal.pone.0284210)

Graphical abstract


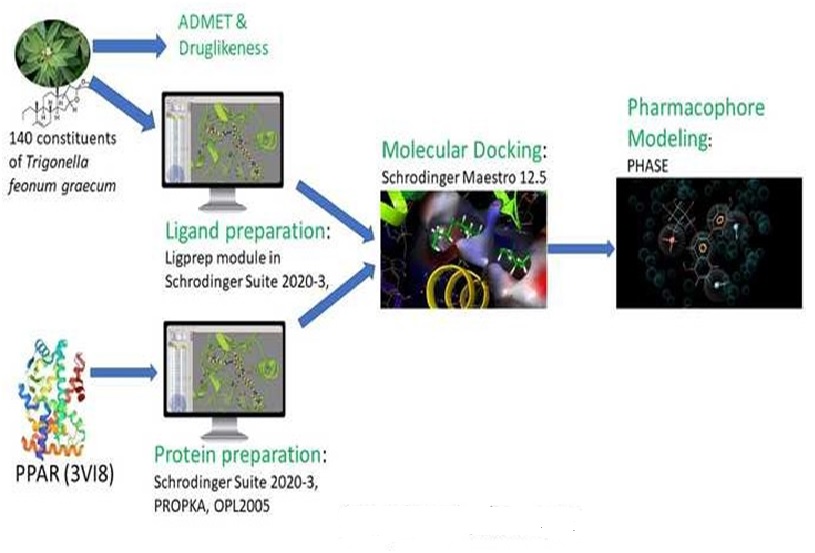

Supplement: S1 Graphical abstract — (DOCX) [file pone.0284210.s001.docx]
